# Impact of Weekly Physical Activity on Stress Response: An Experimental Study

**DOI:** 10.3389/fpsyg.2020.608217

**Published:** 2021-01-12

**Authors:** Ricardo de la Vega, Ruth Jiménez-Castuera, Marta Leyton-Román

**Affiliations:** ^1^Department of Physical Education, Sport and Human Movement, Autonomous University of Madrid, Madrid, Spain; ^2^Didactic and Behavioral Analysis in Sport Research Group, Faculty of Sport Sciences, University of Extremadura, Cáceres, Spain; ^3^Sport of Studies Center, Rey Juan Carlos University, Madrid, Spain

**Keywords:** central fatigue, omega wave, cognitive response, psychophysiology, stress

## Abstract

The aim of this research is focused on analyzing the alteration of the psychophysiological and cognitive response to an objective computerized stress test (Determination Test - DT-, Vienna test System^®^), when the behavioral response is controlled. The sample used was sports science students (N = 22), with a mean age of 22.82 (M_age_ = 22.82; *SD*_years_ = 3.67; M_PhysicalActivity hours/Week_ = 7.77; *SD*_hours_/_week_ = 3.32) A quasi-experimental design was used in which the response of each participant to the DT test was evaluated. The variable “number of hours of physical activity per week” and the variable “level of behavioral response to stress” were controlled. Before and after this test, the following parameters were measured: activation and central fatigue (Critical Flicker Fusion Threshold (CFF Critical flicker fusion ascending and Critical flicker fusion descending; DC potential), and perceived exertion (Central Rating of Perceived Exertion and Peripheral Rating of Perceived Exertion). Significant differences were found in all of the measures indicated. The usefulness of this protocol and the measures used to analyze the stress response capacity of the study subjects are discussed.

## Introduction

The analysis of psychophysiological fatigue is considered very important in different contexts (Lohani et al., 2019). In this sense, the consideration of the study of humans’s response to external and internal loads (Wijesuriya et al., 2007; Wilson et al., 2007) has become one of the most important research topics. The external loads exerted on the individual are added to their skills and coping strategies, resulting in a level of tolerance and adaptation to each situation (Folkman and Lazarus, 1988). Along the last decades, distinctions are often made between physical and mental fatigue role, indicating clear methodologies for the analysis of physiological fatigue, but with clear limitations in the study of central fatigue, because this is measurable only indirectly, which emphasizes the importance of developing new central fatigue analysis procedures (Bittner et al., 2000).

Throughout the decades of research on this topic, different strategies have been used to evaluate the adaptation to these external and internal loads (Lazarus, 1990; Amann, 2011). Thus, for example, a multitude of self-reports and standardized tests have been used (Britner et al., 2003), to which physiological and biological measures have been added (Arza et al., 2019). However, relatively low attention is usually given to the Central Nervous System (CNS)-related mechanisms, which play a major role on the development of fatigue (Tarvainen et al., 2014), but are rarely monitored in the sport and physical activity field (Valenzuela et al., 2020). Most of the studies related to central fatigue to date have focused on the effect it has on performing strenuous physical tasks (Amann and Dempsey, 2008), although over the last few years there has been a notable increase in interest in studying the role of central fatigue in explaining human performance (Inzlicht and Marcora, 2016). In this sense, the psychobiological model based on motivational intensity theory has gained special strength (Gendolla and Richter, 2010). This model emphasizes that perception of effort and potential motivation are the central determinants of task engagement. Both variables are taken into consideration in our research, controlling the involvement in the task (motivation), by applying a computerized test, and analyzing the perception of both central and peripheral effort as detailed in the methodological section.

Two of these measures, which focus the methodological attention of this research due to its great potential in the study of this topic, are the Critical Flicker Fusion Threshold (CFFT), evaluated using one Flicker Fusion instrument (Vicente-Rodríguez et al., 2020), and the DC Potential, evaluated using the OmegaWave technology. The neuro-physiological basis of flicker perception is complex but well established (Görtelmeyer and Zimmermann, 1982). In particular, flickering light directly influences cortical activity. The CFFT was measured using two red light- emitting diodes in binocular foveal fixation. The continuous psychophysical method of limits was employed to determine CFFT (Woodworth and Schlosberg, 1954). The utility of CFFT in sport has been focused on the relationship of arousal level with CNS (Görtelmeyer and Zimmermann, 1982). Increase in CFFT suggests an increase in cortical arousal and sensory sensitivity. By contrast, a decrease of CFFT suggests a reduction in the efficiency of the system to process information (Li et al., 2004; Clemente and Díaz, 2019). On the other hand, for the evaluation of the brain’s direct current (DC) potentials -slow potentials that reflect alterations in brain excitability- OmegaWave technology has gained strength in recent years (Naranjo-Orellana et al., 2020; Valenzuela et al., 2020). This device not only measures the Heart Rate Variability (HRV) but it also simultaneously a brainwave signal (DC potential) in order to complement the information obtained from HRV to assess the athlete’s functional state (Naranjo-Orellana et al., 2020). DC potentials—frequency ranges between 0 and 0.5 Hz, are correlated with different brain processes, such as take consciousness during decision making (Guggisberg and Mottaz, 2013) high alertness states (Bachmann, 1984), arousal state (Haider et al., 1981), or attention (Rösler et al., 1997).

To date, most studies conducted in the evaluation of central fatigue have shown that the greatest disturbances are produced by tasks that require efforts at maximum speed that involve a large amount of force (Davranche and Pichon, 2005; Clemente and Díaz, 2019). However, there are very few studies that have analyzed central fatigue through controlled analysis of a task that primarily involves central fatigue (Fuentes et al., 2019). In this sense, the aim is to apply a computerized test (DT, Vienna Test System), that allows evaluating people’s tolerance to stress and central fatigue by applying a standardized protocol, in physical activity practitioners. The knowledge in this field is really limited, for this reason we developed the present research with the aim of studying the modifications in CFFT and DC potentials in a sample group of regular physical activity. The first hypothesis establishes that the computerized stress task increases the participants’ perception of central fatigue, while keeping the perception of peripheral fatigue stable. As a consequence, the second hypothesis establishes that differences will be found in the “post” situation in the CFFT measures and in the central physiological indicators, which would indicate a relationship between the subjective and objective measures of central fatigue.

## Materials and Methods

This study followed a quasi-experimental design (Montero and León, 2007) and it received the approval of the University Ethical Commission in compliance with the Helsinki Declaration. All subjects were informed about the procedure and gave their written consent to participate. This study was carried out complying with the Standards for Ethics in Sport and Exercise Science Research (Harriss et al., 2019).

### Participants

The participants included 22 individuals from Madrid (Spain), 18 of whom were male and 4 females. These participants were aged between 18 and 36 years (*M*_years_ = 22.82, *SD*_years_ = 3.67). All of the participants regularly engaged in physical activity, between 4 and 14 h per week (*M*_hours_/_week_ = 7.77, *SD*_hours_/_week_ = 3.32). The inclusion criteria was that they performed physical activity at least 3 times a week and 150 min of moderate/vigorous physical activity. The exclusion criteria was not correctly performing the proposed measurements. Four participants were excluded from the study for not completing the measurements correctly. Intentional sampling methods were used (Montero and León, 2007). Due to the impossibility of continuing with the data collection due to the Alert State decreed by the Spanish Government as a result of COVID-19, the sample had to be closed with the participants who had passed all the tests before March 2020.

### Instrumentation and Study Variables

The number of hours of physical activity per week and the scores obtained on the DT test were used as controlled variables. This allows us to know that the differences found are not due to the ability to respond to stress, or to the weekly amount of physical exercise performed. Therefore, only the subjects in which there were no statistically significant differences in their weekly level of physical exercise, nor in the scores obtained in the DT test, were used.

To carry out this research, three measurement systems have been used: OmegaWave device, Flicker Fusion Unit (Vienna Test System), and the Determination Test (Vienna Test System). OmegaWave is a device that assesses the physiological readiness of athletes by examining the autonomic balance through HRV and brain‘s energy balance via DC potential (Gómez-Oliva et al., 2019), Elastic chest band MEDITRACE (dominant hand and forehead). Coach + application (OmegaWave Ltd, Espoo, Finland) was used on Ipad mini 2 32GB. The Vienna Test System is an instrument for computerized psychological assessments that allows the objective evaluation of different psychological parameters. The Determination Test (DT Vienna test system) (Whiteside, 2002; Whiteside et al., 2003) was used to determine neuropsychological fatigue. The test studied the attentional capacity, reactive stress tolerance, reaction speed among continuously, and quickly changing acoustic and visual stimuli. The test is simple, the difficulty of the task lies in the different modality of the arriving stimuli and their speed. This way we measure those cognitive abilities of the people involved that are needed for the distinction of colors and sounds, the perception of the characteristics of stimuli, their memorization, and finally, the selection of the adequate answer. The stimuli coming during the test are not predictable. Instead, the subjects need to react to them randomly (Schuhfried, 2013). We study four key variables: the average value of reaction speed (sec), the number of correct answers (raw score), which reflects the ability of the respondent to precisely and quickly select the adequate answer even under pressure. Furthermore, we also examine the number of incorrect answers (raw score) which can show us how likely the respondent is to get confused under stress and pressure; finally, the high number of missed answers (raw score) reveals that the respondent is not capable of maintaining his/her attention under stress and is prone to giving up these situations (Neuwirth and Benesch, 2012). The duration of this test was 6 min.

Before and after the stress test the following parameters were analyzed in this order:

Parameters analyzed through OmegaWave Coach + device^®^ (OmegaWave Ltd, Espoo, Finland):

– Hear Rate Variability (HRV). Square root of the mean of the squares of successive RR interval differences (RMSSD), Standard deviation of all normal to normal RR intervals (SDNN), and Standard deviation of successive squares of intervals RR (SDSD). OmegaWave is a device that assesses the physiological readiness of athletes by examining autonomic balance through HRV and brain‘s metabolic state via DC potential (Ilyukhina and Zabolotskikh, 2020). Elastic chest band MEDITRACE (dominant hand and forehead). Coach + application (Omegawave Ltd., Espoo, Finland) was used on Ipad mini 2 32GB. For calculating HRV it be used the Root Mean Square of the Successive Differences score (RMSSD) (Ilyukhina et al., 1982). It was used before and after the stress test.
– DC potential dynamics. DC Potential represent changes in the brain’s metabolic balance in response to increased exercise intensity or psychological challenges and are linked to cognitive and mental load (Wagshul et al., 2011; Ilyukhina, 2015).
– CNS System Readiness (Ilyukhina, 1986). It’s indicated by a floating grade from 1.0 to 7.0, where 7.0 is the optimal state. This index represents the state of the brain’s energy level and is composed of three factors (in order of significance): stabilization point of DC potential (mV), stabilization time (reduces system readiness state of 1.0–7.0, if not optimal), and curve shape (reduces system readiness state of 1.0–7.0, if not optimal).
– Stabilization point of DC Potential (mV) (Ilyukhina et al., 1982; Ilyukhina, 2013): The first priority in DC analysis is the stabilization point of DC Potential. In the literature, especially by Ilyukhina, this point is defined as Level of Operational Rest. In 1982, the combined work of Ilyukhina and Sychev was published which outlined quantitative parameters of LOR for the assessment of the healthy human’s adaptation and compensatory−adaptive abilities to physical and mental loads in sports.
– Stabilization time (Ilyukhina and Zabolotskikh, 1997). The second priority of analysis is to look at the stabilization time. measured in minutes. The spontaneous relaxation speed represents neuroreflex reactivity (neural control of baroreflex arch) of cardiovascular and respiratory systems. This measure associated with psycho-emotional dynamic and stability. Normal stabilization time occurs within 2 min and represents optimal balance within stress-regulation systems.
– Curve Shape: The curve shape is composed of two elements: Difference between measurement start mV and end mV values (Table 1). The optimal shape of the curve should show a smooth transition from a higher initial value (active wakefulness) to a lower stabilization value (operational rest DC potential form represents the dynamic interaction within stress-regulation systems). DC potential form can indicate the level of CNS activation balance.

Parameters analyzed though Flicker Fusion unit (Vienna Test System^®^):

– Critical flicker fusion ascending (Hz) (CFFA) and Critical flicker fusion descending (Hz) (CFFD). Cortical arousal was measured using the critical flicker fusion threshold (Hz) (CFFT) in a viewing chamber (Vienna Test System^®^), following the procedure of previous studies (Clemente et al., 2016). An increase in CFFT suggests an increase in cortical arousal and information processing; a decrease in CFFT values below the baseline reflects a reduction in the efficiency of information processing and central nervous system fatigue (Whiteside, 2002). It was used before and after the stress test.

Parameters analyzed though DT test (Vienna Test System^®^):

– We study four key variables: the average value of reaction speed (msec), the number of correct answers (raw score), which reflects the ability of the respondent to precisely and quickly select the adequate answer even under pressure. Furthermore, we also examine the number of incorrect answers (raw score) which can show us how likely the athlete is to get confused under stress and pressure; finally, the high number of missed answers (raw score) reveals that the respondent is not capable of maintaining his/her attention under stress and is prone to giving up these situations (Neuwirth and Benesch, 2012). The duration of this test was 6 min without instructions.

Parameters analyzed by self-report instruments:

– Central Rating of Perceived Exertion (RPEC) and Peripheral Rating of Perceived Exertion (RPEP). The Rating of Perceived Exertion (Borg, 1998), was used as a measure of central (cardiorespiratory) and peripheral (local-muscular, metabolic) exertion before and after the stress test (Bolgar et al., 2010; Cárdenas et al., 2017). The RPE is a 15 point category-ratio; the odd numbered categories have verbal anchors. Beginning at 6, “no exertion at all,” and goes up to 20, “maximal exertion.” Before testing, subjects were instructed on the use of the RPE scale (Noble and Robertson, 1996). We use the scale with the clear differentiation between central as peripheral perceived exertion following the recommendations of the medical staff and under the guideline of Borg (Borg, 1982), for applied studies.

**TABLE 1:** Simplified curve change mV reduction algorithm.

**Start mV—End mV Diff**	**Grade of reduction to OverallDC**
18.0–45.0 mV	No impact
45.0 mV–55 mV or 7.5–18 mV	Moderate reduction
Below 7.5 mV or more than 55 mV	Significant reduction

### Procedure

The participants were contacted and informed about the measurement protocol and of the date and time of the data collection. All of the measurements were collected during the same day. The total data collection time per participant was approximately 45 min. The order of measurements was the following: CFFT, DC Potential, RPE, DT test, RPE, CFFT, and DC Potential.

### Statistics

Data were analyzed using the Statistical Package for the Social Sciences (SPSS) version 21 (SPSS Inc., Chicago, Ill., United States). Means and SDs were calculated using traditional statistical techniques. Normality was tested with the Shapiro-Wilk test. As the distributions were not adjusted to the normal, non-parametric tests were used. A Wilcoxon sign ranges test for intragroup comparisons were conducted to analyze differences between pre and post-test. A Rho Spearman coefficient was used to know the correlations between variables. The Effect Size was tested using the formula = Z/$\sqrt{N}$ for non-parametric tests (Tomczak and Tomcak, 2014). Following the considerations of Cohen (1988), the effect size is considered small when the value is inferior to 0.10, medium when it varies between 0.10 and 0.30 and high when it is superior to 0.50. The significance level was set at *p* < 0.05.

## Results

### Descriptive Analysis, Normality Test According N, Wilcoxon Test, and Effect Sizes

Firstly, the normality tests were realized with the Shapiro-Wilk test. It was determined that most of the variables were not normal, due to which non-parametric statistical tests were applied. In relation to the descriptive analyzes of the study variables, shown in Table 2, after applying the stressor via the DT test, worse values were obtained in all the variables measured. This reflects the alterations in the central response evaluated. Regarding the Wilcoxon rank test that was used to analyze whether there were differences between the scores obtained before and after applying the stressor (DT test), significant differences were found in the variables OverallDc (*p* < 0.05), Flicker ascending (*p* < 0.01), Flicker descending (*p* < 0.01), Central RPE (*p* < 0.01) and Physical RPE (*p* < 0.01), while not finding significant differences in the rest of the variables (Table 2).

**TABLE 2:** Descriptive analysis of the measured variables.

**Variables**	**M^a^**	**SD^b^**	**S-W^c^**	**Z^d^**	**Sig**	**Effect size**
OverallDC (pre-test)	3.62	1.32	0.17	–2.21	0.02	0.47
OverallDC (post-test)	3.07	1.37	0.36			
CNS System (pre-test)	4.68	1.29	0.04	–1.20	0.23	0.25
CNS System (post-test)	4.36	1.17	0.00			
Stabilization DC (pre-test)	7.84	8.59	0.03	–1.83	0.06	0.39
Stabilization DC (post-test)	5.42	10.60	0.01			
Stabilization Time (pre-test)	154.52	49.23	0.00	–0.34	0.74	0.07
Stabilization Time (post-test)	157.75	52.86	0.00			
Curve Shape (pre-test)	6.06	1.25	0.00	–0.41	0.68	0.08
Curve Shape (post-test)	5.93	1.29	0.00			
CFFA (pre-test)	36.63	2.88	0.00	–3.72	0.00	0.79
CFFA (post-test)	38.05	2.89	0.01			
CFFD (pre-test)	38.805	2.64	0.04	–2.37	0.01	0.50
CFFD (post-test)	37.99	3.38	0.03			
RPEC (pre-test)	9.55	2.13	0.25	–4.11	0.00	0.88
RPEC (post-test)	14.55	2.22	0.36			
RPEP (pre-test)	9.18	2.32	0.13	–3.56	0.00	0.76
RPEP (post-test)	10.82	2.40	0.04			
RMSSD (pre-test)	62.29	28.35	0.94	–0.34	0.73	0.07
RMSSD (post-test)	61.02	24.12	0.83			
SDNN (pre-test)	71.15	26.11	0.04	–1.05	0.29	0.22
SDNN (post-test)	68.04	24.77	0.01			
SDSD (pre-test)	78.42	35.62	0.10	–0.44	0.66	0.09
SDSD (post-test)	76.93	30.68	0.82			
DT_MTR	0.70	0.05	0.48			
DT_CR	551.23	44.79	0.11			
DT_IR	38.50	30.57	0.00			
DT_O	24.36	13.28	0.23			

*^*a*^Media. ^*b*^Standard Deviation. ^*c*^Shapiro–Wilks. ^*d*^Wilcoxon sign ranges test.*

### Correlation Analysis

A Spearman bivariate correlation analysis was performed. Spearman’s Rho coefficient was used, since the distribution was non-parametric. Note that significant correlations were found (Table 3) entre OverallDC con DCSSatabilizationLevel (*p* = 0.000; *r* = 0.791^∗∗^); OWCNS (*p* = 0.005; *r* = 0.581^∗∗^); OWDCC (*p* = 0.013; *r* = 0.522^∗^); Flicker Descending (*p* = 0.044; *r* = 0.432^∗^). DCSStabilizationLevel con OWCNS (*p* = 0.000; *r* = 0.766^∗∗^); Flicker Descending (*p* = 0.049; *r* = 0.424^∗^). DCSStabilizationTime con OWCNS (*p* = 0.005; r = 0.572^∗^); OWDCC (*p* = 0.046; *r* = 0.430^∗^); Flicker Ascending (*p* = 0.006; *r* = 0.563^∗∗^). OWCNS correlated with Flicker Ascending (*p* = 0.018; *r* = 0.499^∗^), and SDSD with Flicker Descending score (*p* = 0.046; *r* = −0.430^∗^).

**TABLE 3:** Rho Spearman coefficient.

**Variables**	**1**	**2**	**3**	**4**	**5**	**6**	**7**	**8**	**9**	**10**	**11**	**12**
OverallDC	1.00	0.791^**a^	0.58**	0.40	0.52^*b^	0.36	0.43*	–0.16	0.08	0.10	0.04	0.85
CNS System	0.58**	0.76**	1.00	0.57**	0.15	0.49**	0.27	–0.26	–0.07	0.11	0.01	0.10
Stabilization DC	0.791**	1.00	0.76**	0.32	0.11	0.22	0.42*	–0.29	–0.02	0.08	0.05	0.07
Stabilization Time	0.40	0.32	0.57**	1.00	0.43*	0.56**	0.38	0.07	0.13	–0.09	–0.14	–0.13
Curve Shape	0.52*	0.11	0.15	0.43*	1.00	0.27	0.06	0.13	0.19	0.12	–0.05	0.09
CFFA	0.36	0.22	0.49**	0.56**	0.27	1.00	0.39	–0.18	0.17	–0.12	–0.18	–0.13
CFFD	0.43*	0.42*	0.27	0.38	0.06	0.39	1.00	–0.16	–0.03	–0.41	–0.41	−0.43*
RPEC	–0.16	–0.29	–0.26	0.07	0.13	–0.18	–0.16	1.00	0.41	0.01	0.18	0.00
RPEP	0.08	–0.02	–0.07	0.13	0.19	0.17	–0.03	0.41	1.00	–0.42	–0.29	–0.41
RMSSD	0.10	0.08	0.11	–0.09	0.12	–0.12	–0.41	0.01	–0.42	1.00	0.89**	0.99**
SDNN	0.04	0.05	0.01	–0.14	–0.05	–0.18	–0.41	0.18	–0.29	0.89**	1.00	0.87**
SDSD	0.85	0.07	0.10	–0.13	0.09	–0.13	−0.43*	0.00	–0.41	0.99**	0.87**	1.00

^a*^p < 0.01. ^b**^p < 0.05.

## Discussion

The objective of the present research was to study the modification of DC potentials and the CFFT scores after the computerized stress test (DT). The analysis of the subjective cognitive responses about fatigue after DT test reveals significant differences in the participants, both at a physical and central level. As regards the first hypothesis, it is partially fulfilled. There are significant differences in central perceived fatigue, with a very high effect size, which supports the hypothesis and emphasizes the usefulness of the established research protocol. However, significant differences also appear in peripheral perceived fatigue, which is beyond the initial approaches. This result is of special interest because it allows to consider the relationship between both types of perceived fatigue (Bittner et al., 2000; Clemente et al., 2016). These results, taking into account that the participants did the test sitting down, emphasize the effect achieved through the protocol used to generate stress in them, without significant differences in the performance achieved in the task. Previous research carried out with the DT test already points in this same direction (Ong, 2015). The differences found in the perception of physical fatigue even without previous movement are interesting. Similar results are found in studies carried out in contexts such as chess (Fuentes et al., 2019), where central fatigue due to the demands of each game also leads to physical fatigue of the players. This fact seems relevant insofar as the studies should incorporate measures of both dimensions to be able to explain a higher percentage of variance of the results found.

As regards the second hypothesis, the decrease of CFFD values indicates that it has a negative effect generating central fatigue and an alteration in cortical activation (Li et al., 2004; Clemente, 2016). These results confirm the alterations in cortical activation found in physiological efforts of high intensity and of short duration, such as sprints at maximum speed (Clemente et al., 2011). This same trend is also observed in research focused on generating a high level of stress in soldiers, which emphasizes the usefulness of using the DT test to create stress in the participants (Clemente et al., 2016). In line with the ideas defended by Clemente (2016), decreased in CFFD scores seem to be linked to high sympathetic autonomous nervous system activation, which could also affect higher cognitive functions, such as executive processes (i.e., making complex decisions, memory, and attention processes) (Shields et al., 2016). These same considerations can also be made with respect to the significant differences found in CFFA scores. Higher scores are found after the stress test, which implies that the participants have needed more time to respond to the flicker task as consequence of central fatigue (Fuentes et al., 2019; Lohani et al., 2019).

Regarding the results obtained in the Overall DC scores, the significant differences show a pattern of alteration as a consequence of the stress test. As Naranjo-Orellana et al. (2020) point out, the OW test obtains good reliability and validity values using the heart rate variability as a measure in conjunction with the DC Potential (stabilitation DC, stabilitation time, and curve shape). Changes in the DC potentials have been reported to be reflective of performance in different brain processes (Haider et al., 1981; Valenzuela et al., 2020). The lower scores obtained after the stress test could indicate, as with the CFF scores, an increase in central fatigue detected by the OmegaWave system (Valenzuela et al., 2020). This result, in any case, needs to be analyzed in detail in future research.

Therefore, monitoring the DC potentials and the CFF scores could be useful to control the cognitive load of the different tasks that having a high mental demand.

Due to the exceptional circumstances of data collection in the present study, some of the study limitations were the sample size and the small number of women who participated in it. Future research works should expand the sample power, as well as determine its effect in a sedentary sample.

## Conclusion

To conclude, this is the first study that has jointly analyzed the scores obtained in the analysis of low-frequency brain waves (DC potentials), together with those obtained in the Flicker test. In this sense, although the performance in a specific task seems similar, the demand it has for the person must be evaluated, being useful the use of research protocols similar to the ones we have used. The results open a new field where both measurements could be interesting and useful to assess the cognitive demands of persons.

## Data Availability Statement

The raw data supporting the conclusions of this article will be made available by the authors, without undue reservation, to any qualified researcher.

## Ethics Statement

The studies involving human participants were reviewed and approved by the University Ethical Commission in compliance with the Helsinki Declaration. The patients/participants provided their written informed consent to participate in this study.

## Author Contributions

RV: conceptualization, investigation, resources, writing—review and editing, and project administration. RV, ML-R, and RJ-C: methodology, data curation, writing—original draft preparation, visualization, supervision, and formal analysis. ML-R and RJ-C: software and validation.

## Conflict of Interest

The authors declare that the research was conducted in the absence of any commercial or financial relationships that could be construed as a potential conflict of interest.
